# Galangin sensitizes TRAIL-induced apoptosis through down-regulation of anti-apoptotic proteins in renal carcinoma Caki cells

**DOI:** 10.1038/srep18642

**Published:** 2016-01-04

**Authors:** Min Ae Han, Dong Hee Lee, Seon Min Woo, Bo Ram Seo, Kyoung-jin Min, Shin Kim, Jong-Wook Park, Sang Hyun Kim, Yung Hyun Choi, Taeg Kyu Kwon

**Affiliations:** 1 Department of Immunology, School of Medicine, Keimyung University, 2800 Dalgubeoldaero, Dalseo-Gu, Daegu 704-701, South Korea; 2 Department of Pharmacology, School of Medicine, Kyungpook National University, Daegu, South Korea; 3 Department of Biochemistry, College of Oriental Medicine, Dong-Eui University, Busan, South Korea

## Abstract

Galangin, bioflavonoids, has been shown anti-cancer properties in various cancer cells. In this study, we investigated whether galangin could enhance TRAIL-mediated apoptosis in TRAIL resistant renal carcinoma Caki cells. Galangin alone and TRAIL alone had no effect on apoptosis, while combined treatment with galangin and TRAIL significantly induced apoptosis in renal carcinoma (Caki, ACHN and A498) but not normal cells (normal mouse kidney cells and human normal mesangial cells). Galangin induced down-regulation of Bcl-2 protein at the transcriptional level via inhibition of NF-κB activation but not p53 pathway. Furthermore, galangin induced down-regulation of cFLIP, Mcl-1 and survivin expression at the post-translational levels, and the over-expression of Bcl-2, cFLIP, Mcl-1 and survivin markedly reduced galangin-induced TRAIL sensitization. In addition, galangin increased proteasome activity, but galangin had no effect on expression of proteasome subunits (PSMA5 and PSMD4). In conclusion, our investigation suggests that galangin is a potent candidate for sensitizer of TRAIL resistant cancer cell therapy.

Tumor necrosis factor-related apoptosis-inducing ligand (TRAIL) is a protein functioning as a ligand that induces the programmed cell death called apoptosis. TRAIL binding to death receptors (DRs; DR4 and DR5) assembles death-inducing signaling complex (DISC) through recruitment of FAS-associated protein with death domain (FADD) and caspase-8^1^. TRAIL has a huge advantage in its selectivity for targeting cancer due to relatively higher expression of death receptors than normal cells^2^. In contrast, normal cells highly express decoy receptor (DcR)1 and DcR2. TRAIL binds to decoy receptors, but these complexes unable to activate apoptotic signal pathway. Therefore, TRAIL has barely toxicity in normal cells. However, cancer cells acquires resistance by down-regulating DRs^3^ and up-regulating anti-apoptotic proteins including cellular FLICE-like inhibitory protein (cFLIP)^4^, anti-apoptotic Bcl2 family proteins, and inhibitor of apoptosis proteins (IAPs)^5^. Therefore, finding TRAIL sensitizers is necessary to overcome TRAIL resistance.

Flavonoids are a family of polyphenolic compounds, which are common components of the human diet. They have been considered a helpful anti-cancer drug as an apoptosis inducer, inhibitor for proliferation and antioxidant in cancer cells^6^. Among them, galangin (GA, 3,5,7-trihydroxyflavone) is abundant in propolis, natural compound produced by honeybee and in rhizome of *Alpinia officinarum*, and widely used in Asia as a herbal medicine^7^. Galangin shows anti-tumorigenic effects in various cancer cells, including colon cancer^8^, human mammary tumor cell^9^, hepatocellular carcinoma cells^10^, melanoma^11^, ovarian cancer cells^12^, promyelocytic leukemia^13^ and prostate cancer cells^14^. Previous reports elucidated that G0/G1 arrest in cell cycle by down-regulation of cyclins D3, E, and A^9^ and apoptosis via the mitochondrial pathway^10^ are mechanisms underlying anti-tumor effect of galangin. Galangin induces autophagy and apoptosis in various concentrations through upregulation of p53 in HepG2 cells^15^. Szliszka E *et al.* report that ethanolic extract of propolis induces TRAIL-induced apoptotic cell death in prostate cancer cells^14^. However, the molecular mechanisms of galangin-induced TRAIL sensitization are not enough to understand. In this studies, we investigated whether galangin sensitize TRAIL-mediated apoptosis in renal carcinoma Caki cells. We found that galangin sensitized TRAIL-mediated apoptosis through down-regulation of anti-apoptotic factors, including Bcl-2, cFLIP, Mcl-1 and survivin.

## Results

### Galangin enhances TRAIL-mediated apoptosis in human renal carcinoma

**Figure 1:**
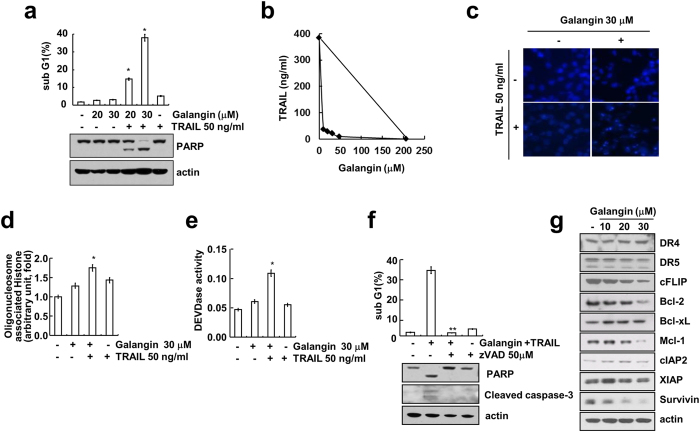
Galangin sensitizes Caki cells to TRAIL-mediated apoptosis. (**a**) Caki cells were treated with 50 ng/ml TRAIL in the presence or absence of the indicated concentrations of galangin for 24 h. The sub G1 population was measured by flow cytometry. The protein levels of PARP were determined by western blot analysis. Actin was used as a loading control. (cropped, full-length blots are in Supplementary Fig. S4). (**b**) Isoboles were obtained by plotting the combined concentrations of each drug required to produce 50% cell death. The straight line connecting the IC_50_ values obtained for two agents when applied alone corresponds to an additivity of their independent effects. Values below this line indicate synergy, whereas values above this line indicate antagonism. (**c–e**) Caki cells were treated with 50 ng/ml of TRAIL with or without 30 μM galangin for 24 h. The condensation and fragmentation of the nuclei were detected by 4’, 6’-diamidino-2-phenylindole staining (**c**). The cytoplasmic histone-associated DNA fragments were determined by a DNA fragmentation detection kit (**d**). Caspase activity was determined with colorimetric assays using caspase-3 (DEVDase) assay kit (**e**). (**f**) Caki cells were treated with 30 μM of galangin and 50 ng/ml of TRAIL for 24 h in the presence or absence of 50 μM z-VAD-fmk (zVAD). The sub G1 population was measured by flow cytometry. PARP and cleaved caspase-3 levels were determined by western blot analysis. Actin was used as a loading control. (cropped, full-length blots are in Supplementary Fig. S4). (**g**) Caki cells were treated with indicated concentrations of galangin for 24 h. The protein level of apoptosis related factors were examined by western blot analysis, such as DR4, DR5, cFLIP, Bcl-2, Bcl-xL, Mcl-1, CIAP2, XIAP and survivin. Actin was used as a loading control. (cropped, full-length blots are in Supplementary Fig. S4). *p < 0.01 compared to the control. **p < 0.01 compared to the co-treatment of galangin and TRAIL.

We examined whether galangin could sensitize TRAIL resistant Caki cells. In FACS analysis, combined treatment with TRAIL and galangin markedly increased sub-G1 population and PARP cleavage in a dose dependent manner, but no increase in treatment with TRAIL alone or galangin alone (Fig. 1A and Supplementary Information Fig. S1). Next, we examined whether combined treatment with galangin and TRAIL have synergistic effects. Galangin plus TRAIL markedly reduced cell viability in various concentrations of galangin and TRAIL. The isobologram analysis suggested that combined treatment with galangin and TRAIL have synergistic effects (Fig. 1B). Combined treatment with galangin and TRAIL caused chromatin damaged in the nuclei (Fig. 1C), and cytoplasmic histone-associated DNA fragmentation (Fig. 1D). Furthermore, combination treatment with galangin and TRAIL induced caspase-2 and 3 activation (Fig. 1E and Supplementary Information Fig. S2), and pan-caspase inhibitor (z-VAD) blocked galangin plus TRAIL-induced apoptosis and cleavage of PARP and caspase-3 (Fig. 1F). Next, we investigated whether the alteration in expression levels of apoptotic regulatory proteins might be associated with galangin-mediated TRAIL sensitization. Western blot analysis showed that expression level of Bcl-2, cFLIP, Mcl-1, and survivin were decreased by galangin in a dose dependent manner. DR4, DR5, Bcl-xL, cIAP2 and XIAP did not alter in response to galangin (Fig. 1G). Taken together, these data indicate that galangin can sensitize Caki cells to TRAIL-mediated apoptosis in a caspase-dependent manner.

### Galangin down-regulates Bcl-2 expression at the transcriptional level through inhibition of NF-κB

**Figure 2:**
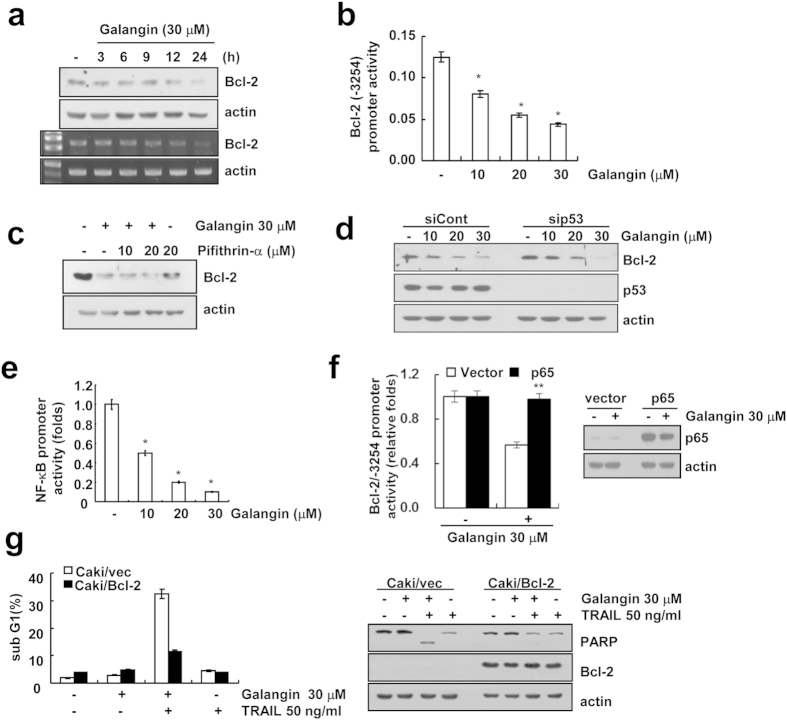
Galangin down-regulates Bcl-2 expression at the transcriptional level in Caki cells. (**a**) Caki cells were treated with 30 μM galangin for the indicated time periods. Protein level and mRNA level of Bcl-2 were examined by western blotting and RT-PCR, respectively. (cropped, full-length blots are in Supplementary Fig. S5). (**b**) Caki cells were transiently transfected with plasmid, Bcl-2 (-3254) bearing the luciferase as a reporter gene under the control of the Bcl-2 promoter. Cells were treated with the indicated concentrations of galangin for 24 h. After lysis of cells, luciferase activity was measured. (**c**) Pifithrin-α was treated with or without galangin to Caki cells. Bcl-2 level was determined by western blot analysis. (cropped, full-length blots are in Supplementary Fig. S5). (**d**) Caki cells were transiently transfected with a control siRNA or p53 siRNA. Twenty-four hours after transfection, cells were treated with the indicated concentrations of galangin for 24 h. Bcl-2 and p53 levels were determined by western blot analysis. (cropped, full-length blots are in Supplementary Fig. S5). (**e**) Caki cells were transiently transfected with a pNF-κB-Luc plasmid containing four copies of the NF-κB binding site, and then treated with the indicated concentrations of galangin for 24 h. After lysis of cells, luciferase activity was measured. (**f**) Caki cells were transiently co-transfected with NF-κB subunit (p65) and Bcl-2-luciferase construct. After transfection, the Caki cells were treated with 30 μM galangin for 24 h. After treatment, the cells were lysed, and the luciferase activity was analyzed. The protein level of p53 was determined by western blot analysis. (cropped, full-length blots are in Supplementary Fig. S5). (**g**) Vector cells harboring empty vector (Caki/vec) and Bcl-2 overexpressing cells (Caki/Bcl-2) were treated with 50 ng/ml TRAIL and 30 μM galangin for 24 h. The sub G1 population was measured by flow cytometry. The protein levels of PARP and Bcl-2 were determined by western blot analysis. Actin was used as a loading control. (cropped, full-length blots are in Supplementary Fig. S5). *p < 0.01 compared to the control. **p < 0.01 compared to the galagin in Caki/vector.

We next explored the underlying mechanisms of galangin-mediated down-regulation of Bcl-2 expression. Expression levels of Bcl-2 protein and mRNA were down-regulated by galangin in a time-dependent manner (Fig. 2A). To examine transcriptional regulation of Bcl-2, Caki cells were transfected with Bcl-2 promoter (Bcl-2/-3254) plasmid. Galangin down-regulated Bcl-2 promoter activity in a dose-dependent manner (Fig. 2B). Previous studies reported that tumor suppressor p53 is a negative regulator of Bcl-2^16^. Thus, we examined whether galangin modulates Bcl-2 transcriptional regulation through p53. We observed that pifithrin-α (p53 inhibitor) and p53 siRNA had no effect on down-regulation of Bcl-2 expression and apoptosis in galangin-treated Caki cells (Fig. 2C,D, and Supplementary Information Fig. S3). Furthermore, galangin did not alter the expression level of p53 protein (Fig. 2D). These data suggested that galangin-induced Bcl-2 down-regulation is not associated with the expression amount of p53. Next, we examined whether galangin inhibits NF-κB-mediated transcriptional activity, since activation of NF-κB induces Bcl-2 mRNA expression^17^. Galangin inhibited NF-κB promoter activity (Fig. 2E). In addition, galangin-mediated inhibitory effect of Bcl-2 promoter was reversed in p65 overexpressed Caki cells (Fig. 2F). To investigate the importance of down-regulation of Bcl-2 expression on galangin plus TRAIL-induced apoptosis, Bcl-2 protein was over-expressed in Caki cells. Overexpression of Bcl-2 markedly inhibited galangin-induced increase of sub-G1 cell population and PARP cleavage (Fig. 2G). These results indicate that NF-κB-dependent and p53-independent down-regulation of Bcl-2 may be involved in galangin plus TRAIL-mediated apoptosis.

### Galangin decreased cFLIP at the post-transcriptional level in Caki cells

**Figure 3:**
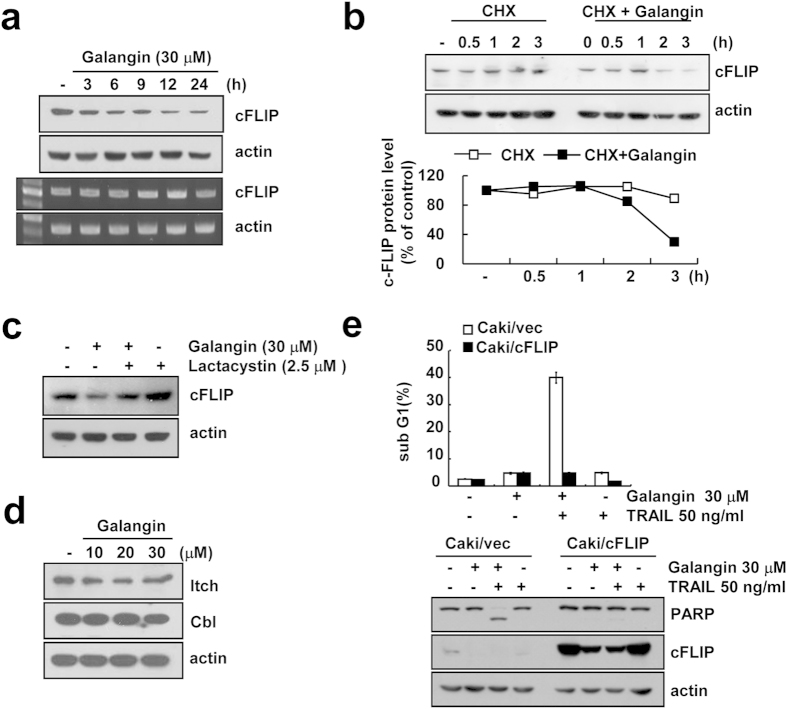
Galangin reduces protein level of cFLIP by post-translational modulation. (**a**) Caki cells were treated with 30 μM galangin for the indicated time periods. Protein level and mRNA level of cFLIP were examined by western blotting and RT-PCR, respectively. Actin was used as a loading control in analysis. (cropped, full-length blots are in Supplementary Fig. S6). (**b**) Caki cells were pretreated with 20 μg/ml cycloheximide (CHX) for 30 min, and then treated with 30 μM galangin for the indicated time periods. Protein level of cFLIP was determined by western blotting. Actin was used as a loading control. The band intensity of cFLIP was measured using the public domain JAVA image-processing program ImageJ. (cropped, full-length blots are in Supplementary Fig. S6). (**c**) Caki cells were pretreated with 2.5 μM lactacystin for 30 min and then treated with 30 μM galangin for 24 h. Protein level of cFLIP was determined by western blotting. Actin was used as a loading control. (cropped, full-length blots are in Supplementary Fig. S6). (**d**) Caki cells were treated with indicated concentrations of galangin for 24 h. Protein levels of Itch and Cbl were determined by western blotting. Actin was used as a loading control. (cropped, full-length blots are in Supplementary Fig. S6). (**e**) Vector cells harboring empty vector (Caki/vec) and cFLIP overexpressing cells (Caki/cFLIP) were treated with 50 ng/ml TRAIL and 30 μM galangin for 24 h. The sub G1 population was measured by flow cytometry. The protein levels of PARP and cFLIP were determined by western blot analysis. Actin was used as a loading control. (cropped, full-length blots are in Supplementary Fig. S6).

Next, we examined the mechanism of cFLIP down-regulation in galagin-treated cells. Since we observed that cFLIP protein but not mRNA level was down-regulated in galangin-treated Caki cells (Fig. 3A), we examined the effect of galangin on the protein stability of cFLIP. Combined treatment with cycloheximide (CHX) and galangin more rapidly degraded cFLIP protein (Fig. 3B). Previous studies reported that cFLIP was mainly degraded by ubiquitn-proteasome pathway^18^. Therefore, we assessed whether ubiquitin-proteasome system is involved in degradation of cFLIP by galangin treatment. Galangin-induced cFLIP down-regulation was inhibited by proteasome inhibitor (lactacystin) (Fig. 3C). cFLIP is degraded via the Cbl and Itch E3 ligase-mediated ubiquitin-proteasome pathways^18^,^19^. Therefore, we examined protein expression levels of Cbl and Itch in galangin treated cells. As shown in Fig. 3D, galangin did not change expression levels of both E3 ligases. Overexpression of cFLIP markedly inhibited galangin-induced increase of sub-G1 cell population and PARP cleavage (Fig. 3E). These data collectively support that down-regulation of c-FLIP by proteasome but not by Cbl and Itch E3 ligase involves galangin plus TRAIL-induced apoptosis.

### Ectopic expression of Mcl-1 and survivin reduces galangin-stimulated TRAIL-mediated apoptosis

**Figure 4:**
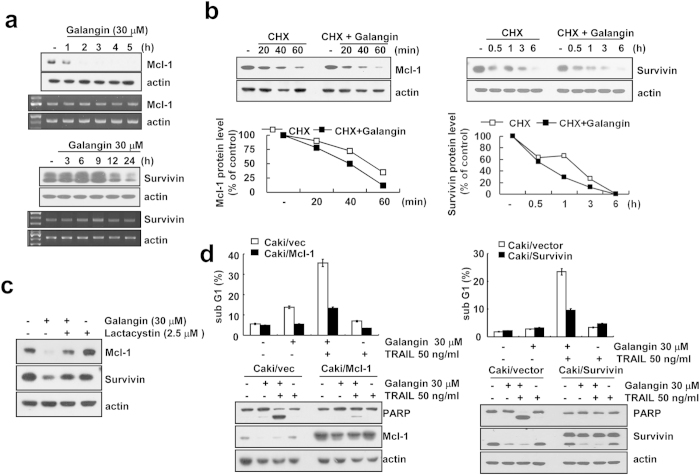
Galangin down-regulates Mcl-1 and survivin protein at the post-transcriptional level in Caki cells. (**a**) Caki cells were treated with 30 μM galangin for the indicated time periods. Protein level and mRNA level of Mcl-1 and survivin were determined by western blotting and RT-PCR, respectivly. Actin was used as a loading control. (cropped, full-length blots are in Supplementary Fig. S7). (**b**) Caki cells were pretreated with 20 μg/ml cycloheximide (CHX) for 30 min, and then treated with 30 μM galangin for the indicated time periods. Protein levels of Mcl-1 and survivin were determined by western blotting. (cropped, full-length blots are in Supplementary Fig. S7). The band intensity of Mcl-1 was measured using ImageJ program. (**c**) After pretreatment with 2.5 μM lactacystin for 30 min, Caki cells were treated with 30 μM galangin for 24 h. Protein levels of Mcl-1 and survivin were determined by western blotting. (cropped, full-length blots are in Supplementary Fig. S7). (**d**) Control empty vector cells (Caki/vec), Mcl-1 overexpressing cells (Caki/Mcl-1) and survivin overexpressing cells (Caki/Survivin) were treated with 50 ng/ml TRAIL and 30 μM galangin for 24 h. The sub G1 population was measured by flow cytometry. The protein levels of PARP, Mcl-1 and survivin were determined by western blot analysis. (cropped, full-length blots are in Supplementary Fig. S7).

Since galangin reduced Mcl-1 and survivin protein expression, as shown in Fig. 1F, we examined the mechanisms of down-regulation of Mcl-1 and survivin in galangin-treated cells. Galangin had no effect on Mcl-1 and survivin mRNA expression (Fig. 4A), whereas galangin induced down-regulation of Mcl-1 and survivin protein expression within 1 and 12 h, respectively. Therefore, we next examined whether galangin modulates the protein stability of Mcl-1 and survivin. As shown in Fig. 4B, CHX gradually decreased Mcl-1 and survivin protein expression, but co-treatment with CHX and galangin more rapidly reduced both proteins expression. To check the ubiquitin-proteasome pathway involved in galangin-mediated down-regulation of Mcl-1 and survivin, Caki cell were treated with proteasome inhibitor. As shown in Fig. 4C, lactacystin restored protein level of Mcl-1 and survivin. These data suggest that galangin induces down-regulation of Mcl-1 and survivin expression at the post-translational level through ubiquitin-proteasome pathway.

To investigate the importance of down-regulation of Mcl-1 and survivin expression on galangin plus TRAIL-mediated apoptosis, Caki cells were treated with TRAIL in the absence or presence of galangin in Mcl-1 and survivin-overexpressing cells, Caki/Mcl-1 and Caki/survvin, respectively. As shown in Fig. 4D, combination treatment with galangin and TRAIL markedly induced apoptosis in Caki/vector cells. However, ectopic expression of Mcl-1 or survivin attenuated induction of apoptosis and PARP cleavage in combined treatment with galangin and TRAIL. Therefore, these data indicated that down-regulation of Mcl-1 and survivin has important roles on galangin-mediated TRAIL sensitization.

### Galangin increases proteasome activity

**Figure 5:**
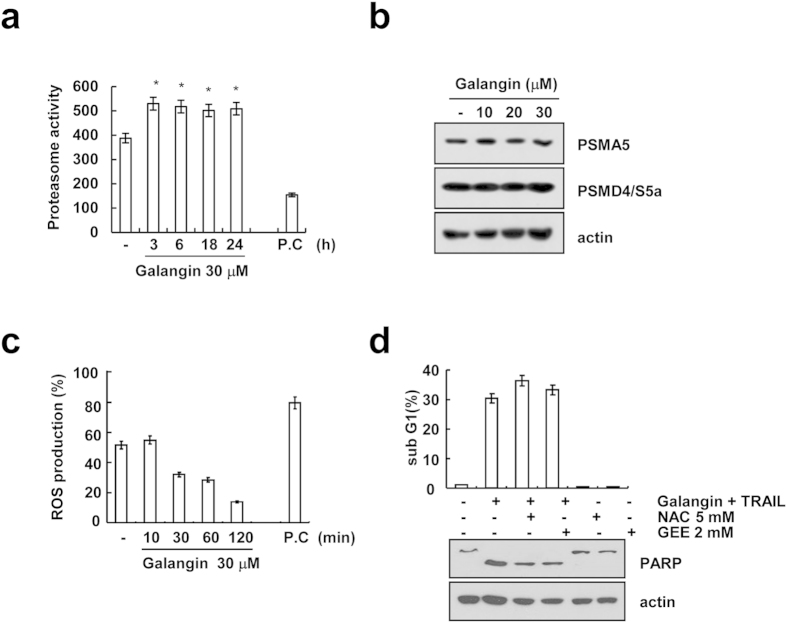
Galangin-induced TRAIL-mediate apoptosis is associated with up-regulation of proteasome activity. (**a**) Caki cells were treated with 30 μM galangin for 24 h. After lysis of cells, the proteasome activity was determined as described in the Materials and Methods. As a positive control, 1 μM MG132 was used. (**b**) Caki cells were treated with indicated concentrations of galangin for 24 h. Protein levels of PSMA5 and PSMD4 were determined by western blotting. Actin was used as a loading control. (cropped, full-length blots are in Supplementary Fig. S8). (**c**) Caki cells were treated with 30 μM galangin for the indicated time periods and loaded with a H_2_DCF-DA fluorescent dye. H_2_DCF-DA fluorescence intensity was detected by flow cytometry. P.C. (Positive control; 20 μM shogaol) (**d**) Caki cells were pretreated with 5 mM NAC and 2 mM GEE for 30 min, and then stimulated with 30 μM galangin plus 50 ng/ml TRAIL for 24 h. Apoptosis was analyzed as the sub G1 population by FACS analysis. The protein expression levels of PARP and actin were determined by Western blotting. The level of actin was used as a loading control. (cropped, full-length blots are in Supplementary Fig. S8).

Galangin reduced c-FLIP, Mcl-1 and survivin at the post-transcriptional levels in a proteasome dependent manner (Figs 3A,C and 4A,C). Therefore, we investigated whether galangin induced proteasome activity. Galangin increased proteasome activity within 3 h, which sustained up to 24 h (Fig. 5A). Therefore, we examined expression levels of two critical proteasome subunits, 26S proteasome non-ATPase regulatory subunit 4 (PSMD4/S5a) and 20 S proteasome subunit alpha type 5 (PSMA5). However, galangin did not alter the expression levels of both proteins (Fig. 5B). Next, we investigated whether reactive oxygen species (ROS) is involved in galangin-mediated TRAIL sensitization. As shown in Fig. 5C,D, galangin did not induce ROS production, and ROS scavengers (NAC and GEE) had no effect on galangin plus TRAIL-induced apoptosis. Therefore, galangin-mediated TRAIL sensitization is independent of ROS signaling.

### Galangin induces TRAIL-mediated apoptosis in other renal cell carcinoma, but not normal cells

**Figure 6:**
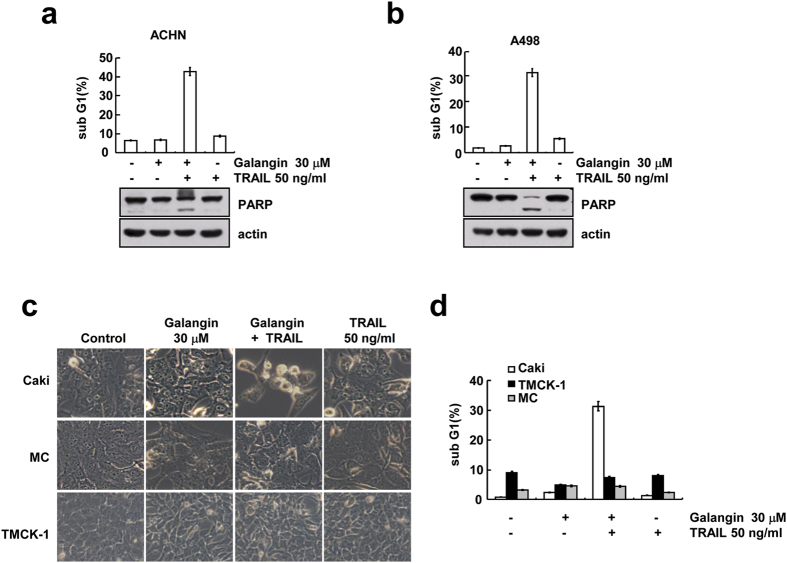
Galangin sensitizes human renal carcinoma cells, but not normal cells to TRAIL-mediate apoptosis. ACHN cells (**a**) and A498 cells (**b**) were treated with 30 μM galangin with or without 50 ng/ml TRAIL for 24 h. The sub G1 population was measured by flow cytometry. The protein levels of PARP were determined by western blot analysis. (cropped, full-length blots are in Supplementary Fig. S9). (**c,d**) Caki, TMCK-1 and mesangial cells (MC) were treated with 50 ng/ml TRAIL with or without 30 μM galangin for 24 h. The morphology of cells was represented using interference light microscopy (**c**). (**d**) The sub G1 population was measured by flow cytometry.

The effect of galagin on TRAIL sensitization was examined in other renal carcinoma (ACHN and A498 cells) and normal cells. As shown in Fig. 6A,B, combined treatment with galangin and TRAIL markedly increased sub-G1 population and PARP cleavage in both cell lines. However, combined treatment with galangin plus TRAIL no produced morphological changes and induction of the sub-G1 population in human mesangial cells (MC) and mouse renal tubular epithelial (TMCK-1) cells (Fig. 6C,D). These data suggest that galangin enhances TRAIL-mediated apoptosis in renal cancer cells, but not in normal cells.

## Discussion

To overcome TRAIL resistance in various cancer cells, identification of sensitizer for TRAIL is required for the establishment of more effective TRAIL-based cancer therapies. In this study, we show that galangin sensitizes Caki cells to TRAIL-mediated apoptosis through down-regulation of Bcl-2, cFLIP, Mcl-1 and survivin expression. Down-regulation of Bcl-2 expression is probably associated with inhibition of NF-κB transcriptional activity. In addition, galangin increased proteasome activity, and induced down-regulation of cFLIP, Mcl-1 and survivin expression at the post-translational levels. Furthermore, galangin enhanced TRAIL-induced apoptosis in other renal carcinoma, but not normal cells. Therefore, our data suggest that galangin could be an attractive candidate for the TRAIL sensitizer.

Previously several reports have shown that galangin induces apoptosis in various cancer cells by different pathways^20^,^21^,^22^. Galangin inhibits proliferation of hepatocellular carcinoma cells by inducing endoplasmic reticulum stress, activating AMPK and mitochondrial dysfunction via up-regulation of Bax and down-regulation of Bcl-2^20^,^21^,^22^. High concentration of galangin (130 μM) induced autophagy through upregulation of p53 in HepG2 cells^15^. Transcription factor p53 has been shown to negatively regulate Bcl-2 transcription levels^16^. However, in our study, low concentration of galangin (30 μM) did not induce up-regulation of p53 (Fig. 2D). In addition, siRNA-mediated p53 knockdown and pretreatment with pifithrin-α (a p53 inhibitor) did not reverse galangin-mediated down-regulation of Bcl-2 expression (Fig. 2D). These results indicated that galangin down-regulated Bcl-2 expression at the transcriptional levels in a p53-independent manner. Bcl-2 contains two promoter regions in 5′-untranslated region (P1 and P2 promoter). The consensus NF-κB site is located in P2 promoter region^23^. Catz *et al.* reported that Bcl-2 promoter activity was increased 10-fold in the presence of the p50/p65 expression vectors^23^. Galangin markedly inhibited NF-κB transcriptional activity (Fig. 2E). Furthermore, ectopic expression of p65 prevented galangin-induced inhibitory effect of Bcl-2 promoter.

Galangin induced down-regulation of cFLIP, Mcl-1 and survivin expression at the post-translational levels (Figs 3 and 4). These proteins are mainly degraded by ubiquintin-proteasome pathway. We found that proteasome inhibitor (lactacystin) markedly reversed galangin-mediated down-regulation of cFLIP, Mcl-1 and survivin expression (Figs 3 and 4) and proteasome activity was increased by treatment with galangin (Fig. 5). E3 ligase, such as Cbl and Itch, induces ubiquitination of c-FLIP and induces its proteasomal degradation. Chang *et al.* reported that TNFα-mediated JNK activation accelerates turnover of the cFLIP through activation of the E3 ubiquitin ligase Itch^19^. Mcl-1, anti-apoptotic Bcl-2 family members, is regulated at multiple levels, involving transcriptional, post-transcriptional and post-translational processes. Mcl-1 is known to be a short-lived protein^24^. Mcl-1 is modulated by the ubiquitin-proteasome system such as four different E3 ubiquitin-ligases (Mule, SCFβ-TrCP, SCFFbw7 and Trim17) and one deubiquitinase (USP9X)^25^. Recently, Ren *et al.* have suggested that E3 ubiquitin ligases β-TrCP and FBXW7 cooperatively mediates GSK3-dependent Mcl-1 degradation^26^. Survivin protein is tightly post-translationally modified by ubiquitylation and phosphorylation^27^. Survivin is also regulated by XIAP-XAF1 complex^28^. Tecleab *et al.* have reported that depletion of K-Ras promotes proteasome degradation of survivin^29^. Proteasome subunits of PSMA5 and PSMD4/S5a play a critical role in proteasomal activity. However, galangin did not alter expression levels of proteasome subunits and E3 ligases in Caki cells (Figs 3D and 5B). Although we did not prove the regulatory mechanism of galangin in down-regulation of cFLIP, Mcl-1 and survivin, we suggest that galangin could potentially modulate the protein stability. Taken together, our results suggest that galangin sensitizes TRAIL-induced apoptosis through the down-regulation of anti-apoptotic proteins (Bcl-2, cFLIP, Mcl-1 and survivin) expression. Therefore, galangin might be a potential sensitizer for the treatment of TRAIL-resistant renal cancer.

## Materials and Methods

### Cell culture and materials

Human renal carcinoma cell lines, Caki, ACHN, and A498 were obtained from the American Type Culture Collection (Manassas, VA, USA). The mouse kidney cells (TMCK-1) was a gift from Dr. T.J. Lee (Yeungnam University, Korea). Primary culture of human mesangial cells (Cryo NHMC) were purchased from Clonetics (San Diego, CA). The culture medium used throughout these experiments was Dulbecco’s modified Eagle’s medium (DMEM) or RPMI containing 10% fetal bovine serum (FBS), 20 mM HEPES buffer and 100 μg/ml gentamycin. Galangin was purchased from Santa Cruz Biotechnology (CA, USA). The PCR primers were purchased from Macrogen (Seoul, Korea). The recombinant human TRAIL was purchased from KOMA Biotech (Seoul, Korea), and z-VAD-fmk, and N-acetyl-L-cysteine (NAC) were obtained from Calbiochem (San Diego, CA, USA). Cycloheximide, lactacystin, pifithrin-α and glutathione ethyl ester (GEE) were purchased from Sigma Chemical Co. (St. Louis, MO, USA). Anti-Bcl-2 (sc-783), anti-DR4 (sc-7863), anti-Bcl-xL (sc-634), anti-Mcl-1(sc-819), anti-p53 (sc-126), anti-NF-κB p65 (sc-8008) and anti-cIAP2 (sc-7944) antibodies were purchased from Santa Cruz Biotechnology (CA, USA). Anti-DR5 (K0411062) antibody was purchased from KOMA Biotech (Seoul, Korea). Anti-PARP (#9542) antibody, anti-PSMA5 (#2457) and anti-PSMD/S5a (#1244) antibody were obtained from Cell Signaling Technology (Beverly, MA). Anti-c-FLIP (ALX-804-961-0100) antibody was obtained from Enzo life science (Farmington, NY). Anti-XIAP (610762) and anti-survivin (AF886) were purchased from BD Biosciences (Bedford, MA) and R&D System (Minneapolis, MN), respectively. Anti-actin (A5441) antibody was obtained from Sigma (St. Louis, MO).

### Flow cytometry analysis

For flow cytometry, the cells were resuspended in 100 μl of phosphate-buffered saline (PBS), and 200 μl of 95% ethanol was added while the cells were being vortexed. Then, the cells were incubated at 4 °C for 1 h, washed with PBS, resuspended in 250 μl of 1.12% sodium citrate buffer (pH 8.4) with 12.5 μg of RNase and incubated for an additional 30 min at 37 °C. The cellular DNA was then stained by adding 250 μl of a propidium iodide solution (50 μg/ml) to the cells for 30 min at room temperature. The stained cells were analyzed by fluorescent-activated cell sorting on a FACScan flow cytometer to determine the relative DNA content, which was based on the red fluorescence intensity.

### Western blot analysis

For the Western blotting experiments, the cells were washed with cold PBS and lysed on ice in modified RIPA buffer (50 mM Tris-HCl pH 7.4, 1% NP-40, 0.25% Na-deoxycholate, 150 mM NaCl, 1 mM Na_3_VO_4_, and 1 mM NaF) containing protease inhibitors (100 μM phenylmethylsulfonyl fluoride, 10 μg/ml leupeptin, 10 μg/ml pepstatin, and 2 mM EDTA). The lysates were centrifuged at 10,000 × *g* for 10 min at 4 °C, and the supernatant fractions were collected. The proteins were separated by SDS-PAGE electrophoresis and transferred to Immobilon-P membranes. The specific proteins were detected using an enhanced chemiluminescence (ECL) Western blotting kit according to the manufacturer’s instructions.

### Determination of synergy and cell viability assay

The possible synergistic effect of galangin and TRAIL was evaluated using the isobologram method. In brief, the cells were treated with different concentrations of galangin and TRAIL alone or in combination. After 24 h, XTT assay was employed to measure the cell viability using WelCount Cell Viability Assay Kit (WelGENE, Daegu, Korea). In brief, reagent was added to each well and was then measured with a multi-well plate reader (at 450 nm/690 nm). Relative survival was assessed and the concentration effect curves were used to determine the IC_50_ (the half-maximal inhibitory concentration) values for each drug alone and in combination with a fixed concentration of the second agent^30^.

### 4′,6′-Diamidino-2-phenylindole staining (DAPI) for nuclei condensation and fragmentation

To examine cellular nuclei, the cells were fixed with 1% paraformaldehyde on glass slides for 30 min at room temperature. After the fixation, the cells were washed with PBS and a 300 nM 4′,6′-diamidino-2-phenylindole solution (Roche, Mannheim, Germany) was added to the fixed cells for 5 min. After the nuclei were stained, the cells were examined by fluorescence microscopy.

### Asp-Glu-Val-Asp-ase (DEVDase) Activity Assay

To evaluate DEVDase activity, cell lysates were prepared after their respective treatments with TRAIL in the presence or absence of galangin. Assays were performed in 96-well microtiter plates by incubating 20 μg of cell lysates in 100 μl of reaction buffer (1% NP-40, 20 mM Tris-HCl, pH 7.5, 137 mM NaCl, 10% glycerol) containing a caspase substrate [Asp-Glu-Val-Asp-chromophore-p-nitroanilide (DVAD-pNA)] at 5 μM. Lysates were incubated at 37 °C for 2 h. Thereafter, the absorbance at 405 nm was measured with a spectrophotometer.

### Reverse transcription polymerase chain reaction (RT-PCR)

Total RNA was isolated using the TriZol reagent (Life Technologies; Gaithersburg, MD), and the cDNA was prepared using M-MLV reverse transcriptase (Gibco-BRL; Gaithersburg, MD) according to the manufacturers’ instructions. The following primers were used for the amplification of human c-FLIP, Bcl-2, Mcl-1, survivin and actin: cFLIP (sense) 5′-CGG ACT ATA GAG TGC TGA TGG-3′ and (antisense) 5′-GAT TAT CAG GCA GAT TCC TAG -3′; Bcl-2 (sense) 5′-GGT GAA CTG GGG GAG GAT TGT-3′ and (antisense) 5′-CTT CAG AGA CAG CCA GGA GAA-3′; Mcl-1 (sense) 5′-GCG ACT GGC AAA GCT TGG CCT CAA-3′ and (antisense) 5′-GTT ACA GCT TGG ATC CCA ACT GCA-3′; survivin (sense) 5′-CAG ATT TGA ATC GCG GGA CCC -3′ and (antisense) 5′-CCA GAG TCT GGC TCG TTC TCA G -3′; and actin (sense) 5′-GGC ATC GTC ACC AAC TGG GAC-3′ and (anti-sense) 5′-CGA TTT CCC GCT CGG CCG TGG-3′. The PCR amplification was carried out using the following cycling conditions: 94 °C for 3 min followed by 17 (actin) or 25 cycles (c-FLIP, Bcl-2, Mcl-1 and survivin) of 94 °C for 45 s, 58 °C for 45 s, 72 °C for 1 min, and a final extension at 72 °C for 10 min. The amplified products were separated by electrophoresis on a 1.5% agarose gel and detected under UV light.

### Construction of Bcl-2, cFLIP and Mcl-1 stable cells

The Caki cells were stably transfected with pMAX-Bcl-2 (provided by Dr. Rakesh Srivastava, NIH/NIA), pcDNA 3.1-cFLIP, pcDNA 3.1-Mcl-1 or control plasmid pcDNA 3.1 vector using LipofectAMINE2000 as recommended by the manufacturer (Invitrogen Carlsbad, CA). After 48 h of incubation, transfected cells were selected in cell culture medium containing 700 μg/ml G418 (Invitrogen). After 2 or 3 weeks, to eliminate the possibility of clonal differences between the generated stable cell lines, the pooled clones were tested for Bcl-2, cFLIP(L) and Mcl-1 expression by immunoblotting, and the cells were used in this study.

### DNA transfection and luciferase assay

Transient transfection was performed in 6-well plates. One day before the transfection, Caki cells were plated at approximately 60 to 80% confluence. The NF-κB promoter plasmid or Bcl-2/-3254 promoter plasmid was transfected into the cells using Lipofectamine^TM^ 2000 (Invitrogen; Carlsbad, CA). To assess the promoter-driven expression of the luciferase gene, the cells were collected and disrupted by sonication in lysis buffer (25 mM Tris-phosphate, pH 7.8, 2 mM EDTA, 1% Triton X-100, and 10% glycerol), and aliquots of the supernatant were used to analyze the luciferase activity according to the manufacturer’s instructions (Promega; Madison, WI).

### Proteasome activity assay

Chymotryptic proteasome activities were measured with Suc-LLVY-AMC (chymotryptic substrate, Biomol International, Plymouth Meeting, PA). Lysate from galangin-treated cells was prepared. A mixture containing 1 μg cell lysate protein in 100 mM Tris-HCl (pH 8.0), 10 mM MgCl_2_, and 2 mM ATP was incubated at 37˚C for 30 min with 50 μM Suc-LLVY-AMC. Enzyme activity was measured with a fluorometric plate reader at an excitation wavelength of 380 nm and an emission wavelength of 440 nm.

### Measurement of reactive oxygen species (ROS)

Intracellular accumulation of ROS was determined using the fluorescent probes 2′, 7′-dichlorodihydrofluorescein diacetate (H_2_DCFDA). H_2_DCFDA is commonly used to measure ROS generation. Caki cells were treated with galangin, and then cells were stained with the fluorescent dye H_2_DCFDA for an additional 10 min. Then, cells were trypsinized and resuspended in PBS, and fluorescence was measured at specific time intervals with a flow cytometer (Becton–Dickinson; Franklin Lakes, NJ, USA).

### Densitometry

The band intensities were scanned and quantified using the gel analysis plugin for the open source software ImageJ 1.46 (Imaging Processing and Analysis in Java; http://rsb.info. nih.gov/ij).

### Statistical analysis

The data were analyzed using a one-way ANOVA followed by post-hoc comparisons (Student–Newman–Keuls) using the Statistical Package for Social Sciences version 22.0 (SPSS Inc., Chicago, IL, USA).

## Additional Information

**How to cite this article**: Han, M. A. *et al.* Galangin sensitizes TRAIL-induced apoptosis through down-regulation of anti-apoptotic proteins in renal carcinoma Caki cells. *Sci. Rep.* **6**, 18642; doi: 10.1038/srep18642 (2016).

## Supplementary Material

Supplementary Information
