# Supplementary material for: Galangin sensitizes TRAIL-induced apoptosis through down-regulation of anti-apoptotic proteins in renal carcinoma Caki cells
Source: Sci Rep. 2016 Jan 4;6:18642. doi: 10.1038/srep18642 (PMC4698673; doi:10.1038/srep18642)
Supplement: Supplementary Information [file srep18642-s1.pdf]

<Supplementary information>

Galangin sensitizes TRAIL-induced apoptosis through down-regulation of anti-apoptotic proteins in renal carcinoma Caki cells

Min Ae Han[1¶], Dong Hee Lee[1¶], Seon Min Woo[1], Bo Ram Seo[1], Kyoung-jin Min[1], Shin Kim[1], Jong-Wook Park[1], Sang Hyun Kim[2], Yung Hyun Choi[3], Taeg Kyu Kwon[1]*

[1]Department of Immunology, School of Medicine, Keimyung University, 2800 Dalgubeoldaero, Dalseo-Gu, Daegu 704-701, South Korea. [2]Deaprtment of Pharmacology, School of Medicine, Kyungpook National University, Daegu, South Korea. [3]Department of Biochemistry, College of Oriental Medicine, Dong-Eui University, Busan, South Korea.

¶ These authors contributed equally to this work.

* Corresponding author: Taeg Kyu Kwon, Ph.D.
Address: Keimyung University, 2800 Dalgubeoldaero, Dalseo-Gu, Daegu 704-701, South Korea
Tel: 82-53-5803882
E-mail: kwontk@dsmc.or.kr

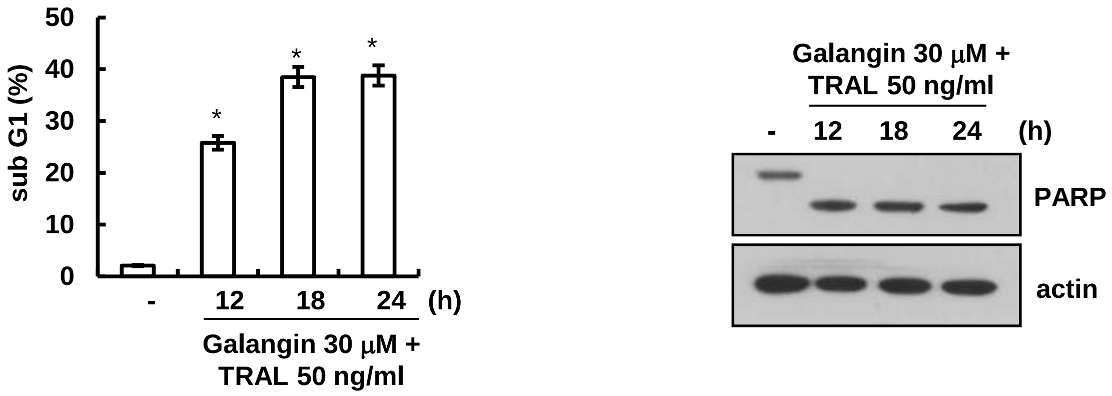

Figure S1. Galangin sensitizes TRAIL-mediated apoptosis in Caki cells. (A) Caki cells were treated with 50 ng/ml TRAIL in the presence or absence of the indicated concentrations of galangin for the indicated time periods. The sub G1 population was measured by flow cytometry (left panel). The protein levels of PARP was determined by Western blot analysis. Actin was used as a loading control (right panel). * $p < 0.01$ compared to the control.

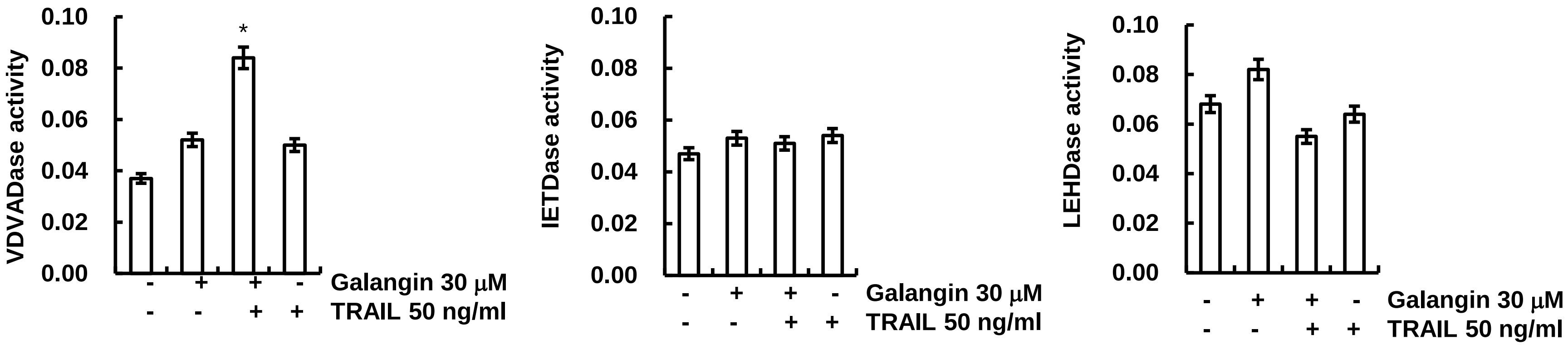

Figure S2. Effect of combined treatment with galangin and TRAIL on caspases activities. Caki cells were treated with 50 ng/ml TRAIL in the presence or absence of 30 μM galangin for 24 h. Caspases activities were determined with colorimetric assays using caspase-2 (VDVADase) assay kits, caspase-8 (IETDase) assay kits and caspase-9 (LEHDase) assay kits. The values in Figure represent the mean ± SD from three independent samples. * $p < 0.05$ compared to the galangin treatment alone.

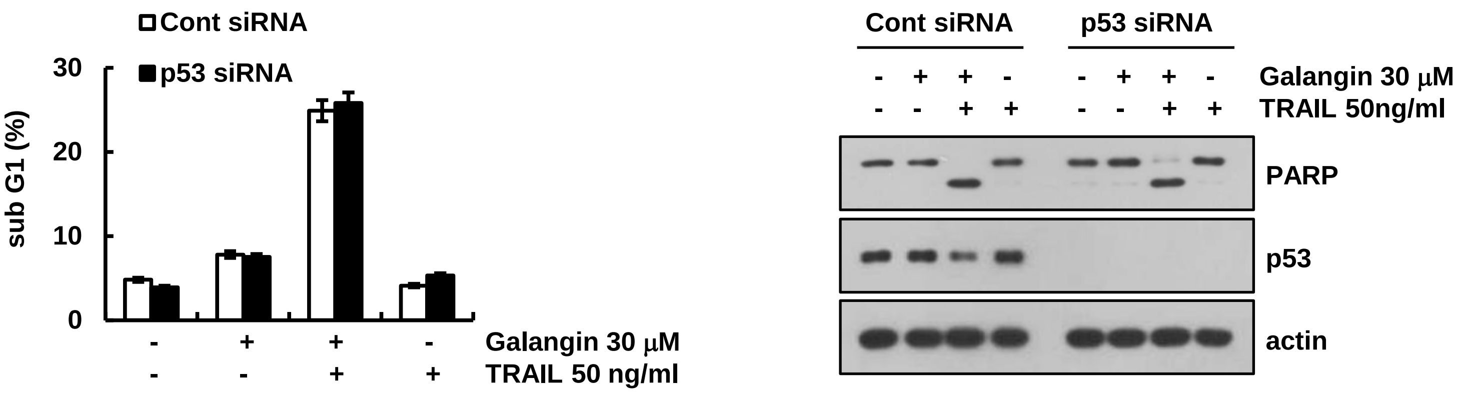

Figure S3. The p53 has no effect on galangin plus TRAIL-induced apoptosis. Caki cells were transiently transfected with a control siRNA or p53 siRNA. Twenty-four hours after transfection, cells were treated with 50 ng/ml TRAIL in the presence or absence of the indicated concentrations of galangin for 24 h. The sub G1 population was measured by flow cytometry (left panel). The protein levels of PARP and p53 were determined by Western blot analysis. Actin was used as a loading control (right panel).

Figure 1a

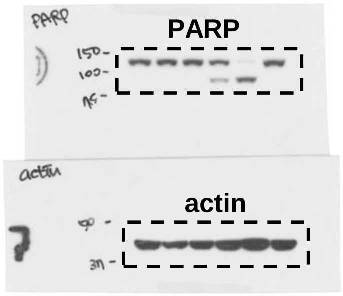

Figure 1f

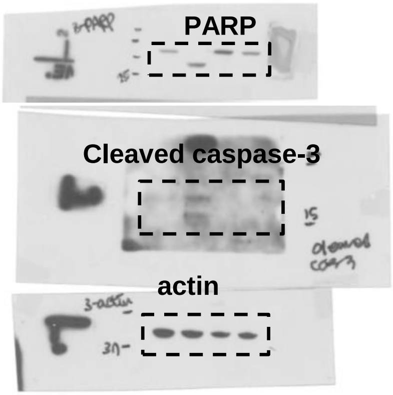

Figure 1g

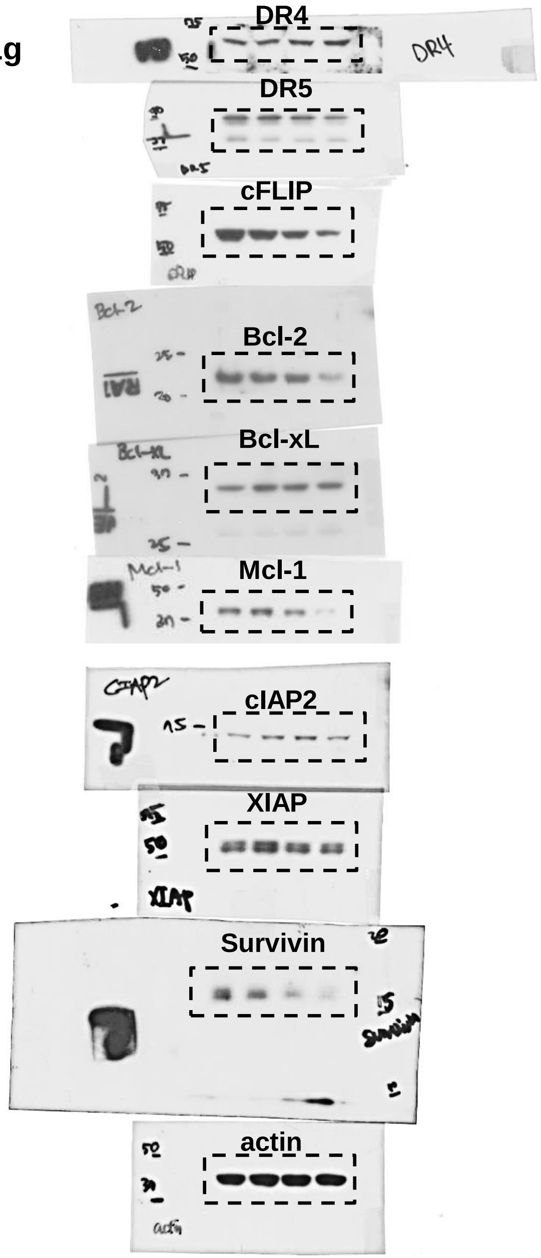

Figure S4. Full-length images of the immunoblots in Figure 1. Black dot line boxes indicate the cropped images used in Figure 1

Figure 2a

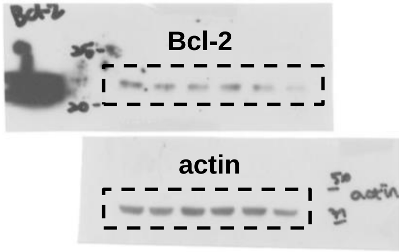

Figure 2c

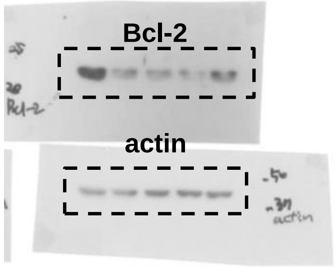

Figure 2d

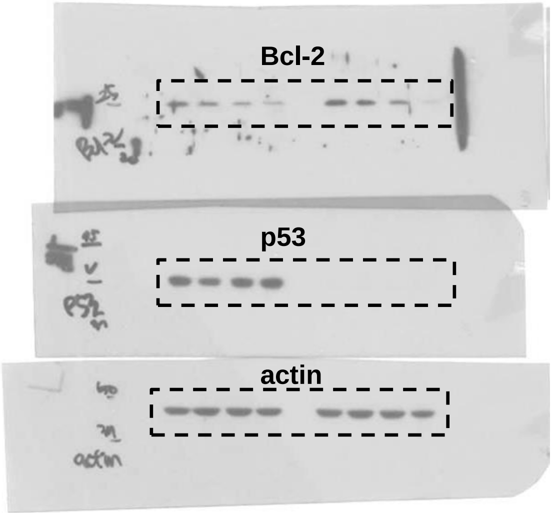

Figure 2f

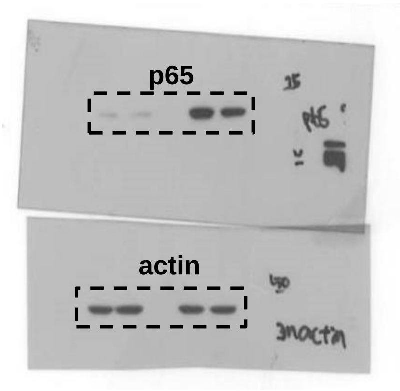

Figure 2g

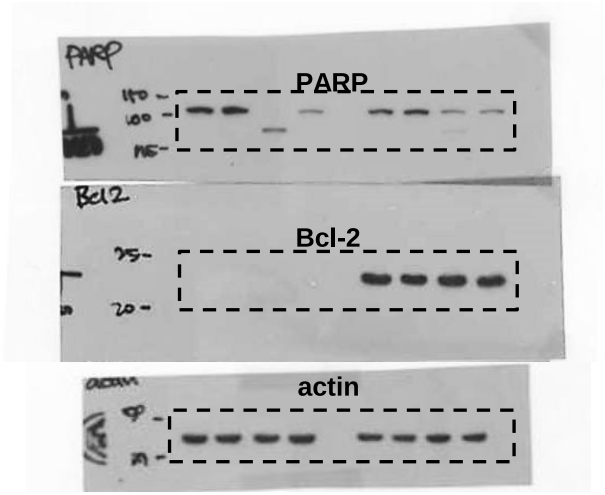

Figure S5. Full-length images of the immunoblots in Figure 2. Black dot line boxes indicate the cropped images used in Figure 2

Figure 3a

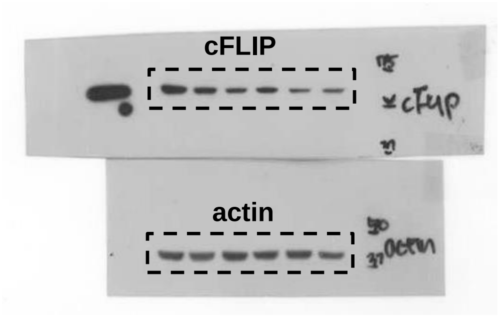

Figure 3b

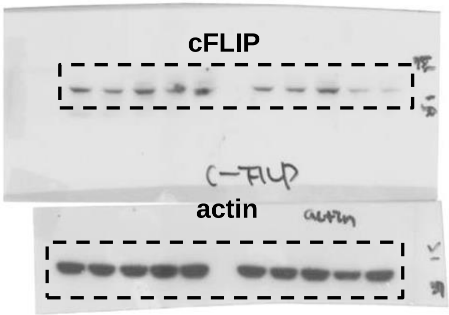

Figure 3c

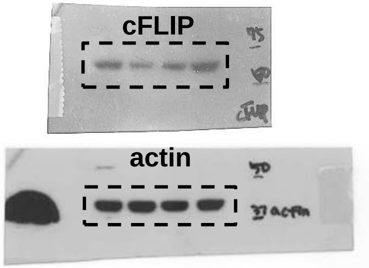

Figure 3d

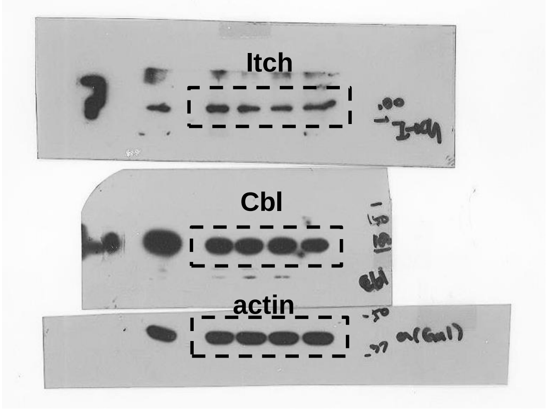

Figure 3e

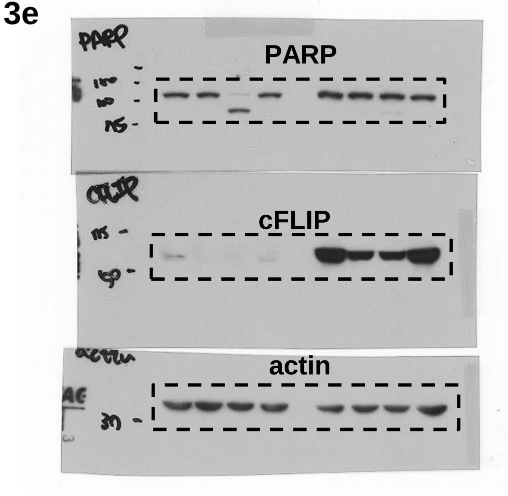

Figure S6. Full-length images of the immunoblots in Figure 3. Black dot line boxes indicate the cropped images used in Figure 3

Figure 4a

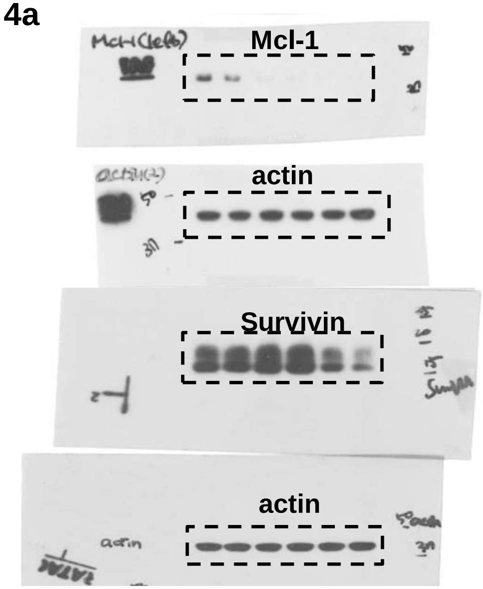

Figure 4b

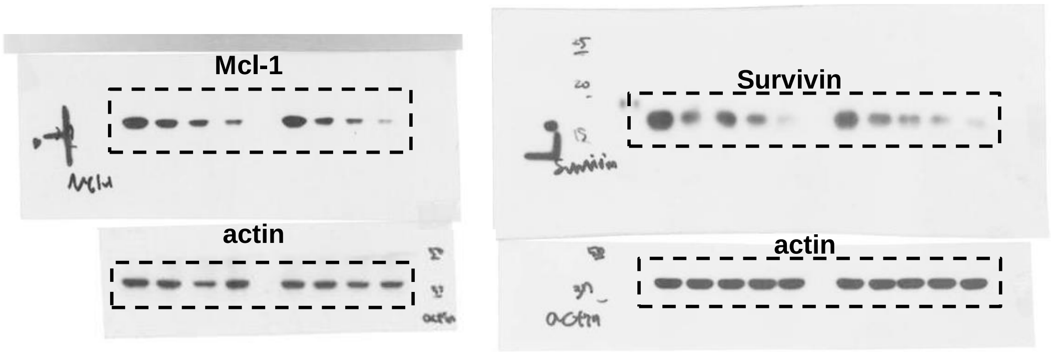

Figure 4c

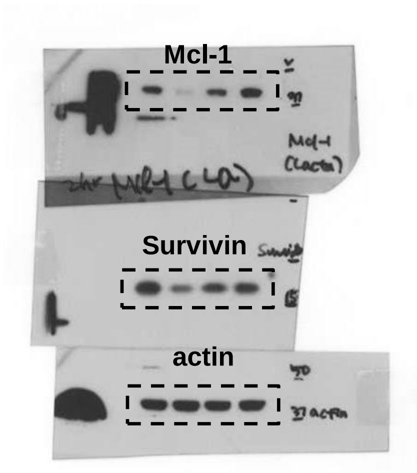

Figure 4d

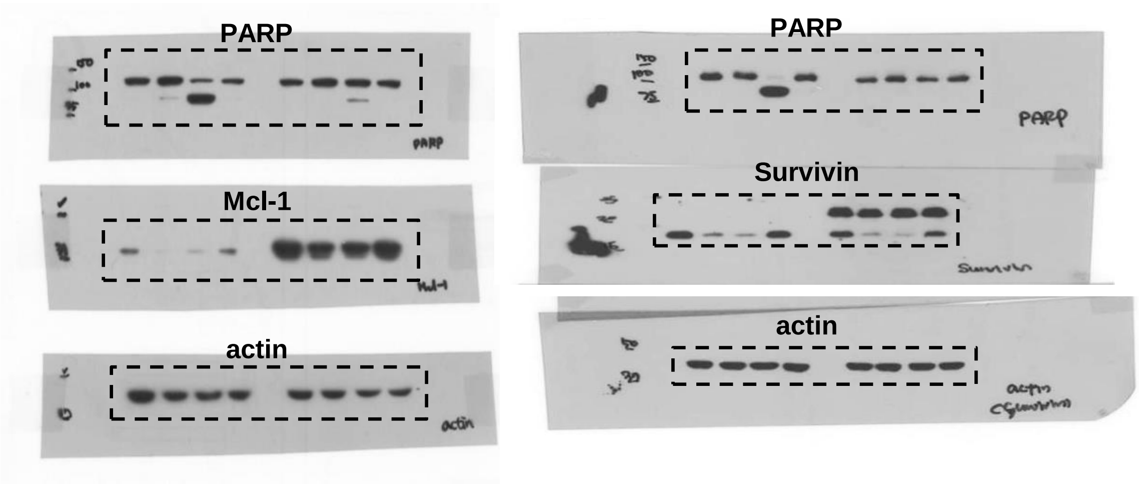

Figure S7. Full-length images of the immunoblots in Figure 4. Black dot line boxes indicate the cropped images used in Figure 4

Figure 5b

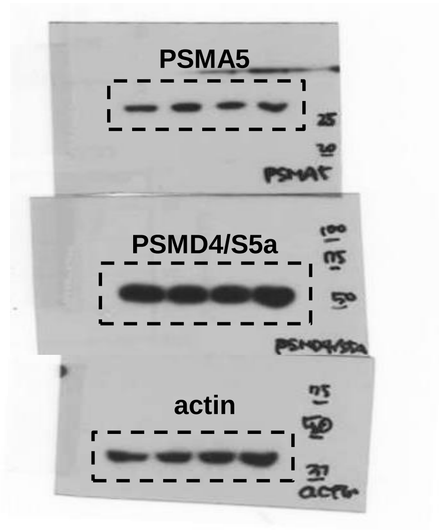

Figure 5d

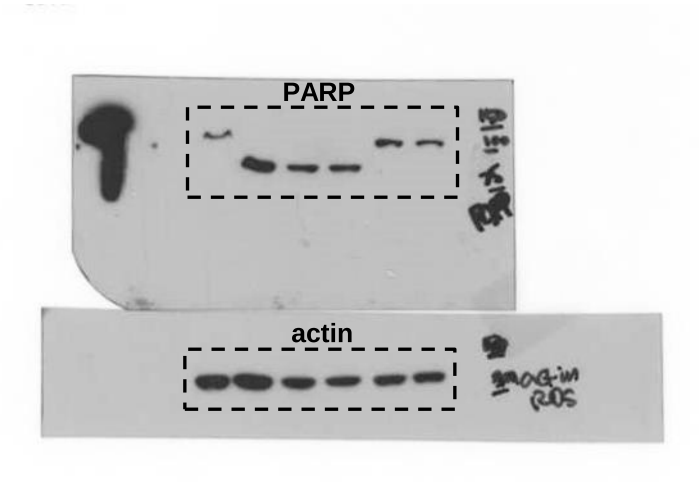

Figure S8. Full-length images of the immunoblots in Figure 5. Black dot line boxes indicate the cropped images used in Figure 5

Figure 6a

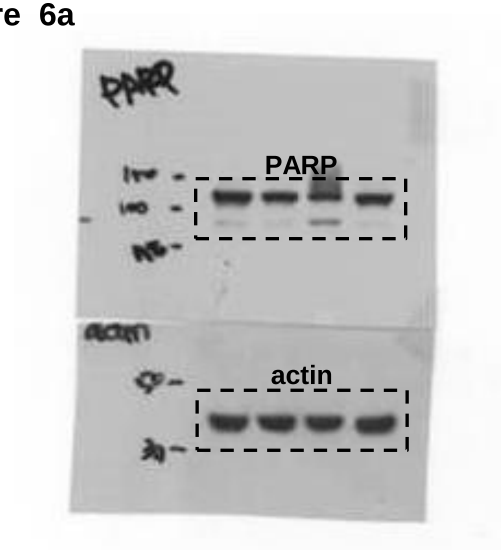

Figure 6b

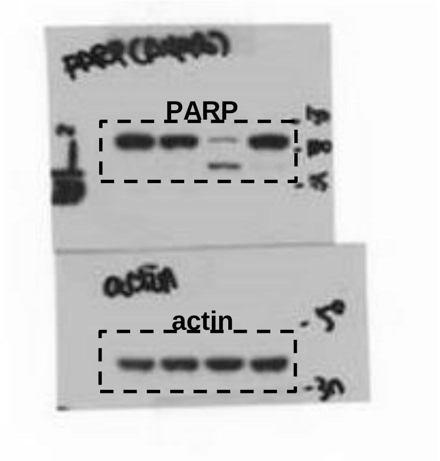

Figure S9. Full-length images of the immunoblots in Figure 6. Black dot line boxes indicate the cropped images used in Figure 6
